# Economic Analysis of the Production of Amylases and Other Hydrolases by *Aspergillus awamori* in Solid-State Fermentation of Babassu Cake

**DOI:** 10.4061/2010/576872

**Published:** 2010-05-23

**Authors:** Aline Machado de Castro, Daniele Fernandes Carvalho, Denise Maria Guimarães Freire, Leda dos Reis Castilho

**Affiliations:** ^1^Renewable Energy Division, Research and Development Center, PETROBRAS, Avenue Horácio Macedo, 950 Ilha do Fundão, Rio de Janeiro 21941-915, Brazil; ^2^COPPE, Chemical Engineering Program, Federal University of Rio de Janeiro, Rio de Janeiro 21949-900, Brazil; ^3^Institute of Chemistry, Federal University of Rio de Janeiro, Rio de Janeiro 21941-909, Brazil

## Abstract

Amylases are one of the most important industrial enzymes produced worldwide, with their major application being in ethanol manufacturing. This work investigated the production of amylases by solid-state fermentation of babassu cake, using the filamentous fungus *Aspergillus awamori* IOC-3914. Lab-scale experiments were carried out to generate input data for simulations of an industrial plant for amylase production. Additionally to the target enzymes, other hydrolases (cellulases, xylanases, and proteases) were also produced, enriching the final product. The most suitable fermentation time was 144 hours, when exoamylase and endoamylase activities of 40.5 and 42.7 U g^−1^ were achieved, respectively. A first evaluation showed a large impact of the inoculum propagation medium on production costs. Therefore, five propagation media were compared, and PDA medium presented the best cost-benefit ratio. The credits obtained from sales of fermented cake as a coproduct enabled a significant decrease in the production cost of the enzyme product, down to 10.40 USD kg^−1^.

## 1. Introduction

Amylases are one of the most important industrial enzymes worldwide [1]. They form an enzyme complex comprising enzymes that act synergistically to break down to glucose the starch polysaccharides amylose, which is composed of linear *α*-1,4-linked glucose units, and amylopectin, which is a glucose-based homopolymer with linear chains and *α*-1,6-linked branches. Thus, amylolytic complexes are formed by three major groups of enzymes: endoamylases, exoamylases, and debranching enzymes: endoamylases (also known as liquefying or depolymerizing enzymes) are composed mainly of *α*-amylases (EC 3.2.1.1) and release oligosaccharides of various lengths by randomly attacking the internal *α*-1,4 linkages. Exoamylases (saccharifying enzymes), composed mostly of glucoamylases (EC 3.2.1.3), release glucose as main product, by cleaving terminal *α*-1,4 bonds. The debranching enzymes, such as pullulanase (EC 3.2.1.41), act predominantly on *α*-1,6 linkages of amylopectin [2, 3]. 

 Within the wide range of applications of amylases, ethanol manufacturing is perhaps the most important one. The two major technologies to convert corn into alcohol are the dry-grind and the wet milling processes. The former is mostly used in the United States and utilizes two different enzyme products that act at medium to high temperatures (60°C and 88°C) and under distinct pH values, thus requiring pH adjustment between the steps of hydrolysis [4]. Cold hydrolysis processes, which consist of raw starch hydrolysis at low temperatures, represent thus an interesting alternative. Although cold hydrolysis was first reported in the 1940s [5], it has just recently been employed commercially at large scale and is currently being pointed out as an outstanding technology [6], which has the prospective of experiencing a 200% market increase up to 2020 [7]. 

 Cold starch hydrolysis presents several advantages over the traditional processes. There are energy savings due to lower temperatures for hydrolysis and simultaneous starch depolymerization and saccharification [7, 8], ethanol yield is higher and byproduct formation is lower [8, 9], water and chemicals consumption is lower, capital costs are decreased, productivity is higher, and less wastes (such as VOC) are generated [10]. 

 However, the costs of enzymes that act under such mild conditions are still high [8]. The absence of the cooking and liquefaction steps, which contribute to release of starch bound to fiber or protein matrixes, is also a disadvantage. Thus, for cold hydrolysis of starch, the addition of accessory enzymes, such as xylanases, cellulases, and proteases, is desired, in order to improve conversion yields [8, 11–13]. 

 Therefore, the development of cost-effective technologies for the production of enzymes for cold starch hydrolysis is of great interest for the ethanol industry. For this purpose, solid-state fermentation (SSF) is a promising technology. Castilho et al. [14] compared the production of lipases using submerged fermentation (SmF) and SSF, reporting that the unitary production cost of those enzymes using SSF is about three times lower than using SmF. Considering SSF, cakes from agroindustry have been described as ideal raw materials for the production of enzymes, since they provide both carbon and nitrogen sources and are usually cheap [15]. 

 Thus, the aim of this work was to investigate the production of amylases and accessory hydrolases with mesophilic characteristics for cold hydrolysis by SSF of babassu cake, as well as to simulate a large-scale process and evaluate its economic performance.

## 2. Materials and Methods

### 2.1. Raw Material

Babassu (*Orbygnia* sp.) cake was kindly provided by TOBASA Bioindustrial de Babaçu S.A. (Tocantinópolis, Brazil). This cake is a subproduct generated in the babassu palm oil extraction industry [16]. The cake was received with a mean particle size of (923 ± 7) *μ*m, which was estimated using a vibratory shaker (Viatest model 76773, Kuhardt, Germany) fitted with sieves from 8 to 150 mesh Tyler. For the solid-state fermentation studies, the cake was dried, grounded, and sieved to obtain particles in the range of 210 to 297 *μ*m (65 and 48 mesh Tyler, resp.).

### 2.2. Microorganism Maintenance and Inoculum Propagation

*Aspergillus awamori* IOC-3914 was obtained from the Instituto Oswaldo Cruz (IOC) culture collection. It was maintained at 4°C in starch agar medium the following (in g L^−1^, anhydrous mass: starch, 10; sodium nitrate, 3; monopotassium phosphate, 1; potassium chloride, 0.5; magnesium sulfate, 0.5; iron sulfate, 0.001; agar, 20) (adapted from [17]). For inoculum propagation, 2.5 × 10^7^ spores from maintenance medium were transferred to each propagation media and incubated for 7 days at 30°C. 

 Five different propagation media were evaluated. Czapeck-Dox medium contained (in g L^−1^, anhydrous mass) (adapted from [18]): sucrose, 30; sodium nitrate, 3; monopotassium phosphate, 1; potassium chloride, 0.5; magnesium sulfate, 0.5; iron sulfate, 0.01; and agar, 13. Malt medium was composed of the following (in g L^−1^, anhydrous mass) (adapted from Farooq et al., 2005): malt extract, 30; peptone, 5; and agar, 20. PDA medium contained the following (g L^−1^) [19]: potato (with hulls), 300 (wet mass); glucose, 15; and agar, 20. Oat medium was composed of (g L^−1^) [20]: oat bran, 50; agar, 30. Finally, starch agar medium (same composition as for strain maintenance above) was also evaluated.

### 2.3. Solid-State Fermentation (SSF) Experiments

Lab-scale experiments were carried out, whereby fungal spores (10^7^ per gram of raw material) were inoculated in tray bioreactors containing 2.5 g of babassu cake. The initial moisture content was adjusted to 70% and the trays were incubated at 30°C. Whole trays were taken daily as samples and submitted to enzyme extraction with distilled water for 30 minutes, at 37°C and 200 rpm, followed by centrifugation for 20 minutes, at 25°C and 11000 g. Supernatants were aliquoted and frozen for further enzymatic quantifications. All experiments were done in duplicate.

### 2.4. Assays

Crude extracts from the fermentation were analyzed regarding their contents of endoamylase, exoamylase, protease, cellulose, and xylanase, which were quantified using substrate solutions (0.5% soluble starch, 1% soluble starch, 0.5% azocasein, 2% carboxymethylcellulose and 1% Birchwood xylan, resp.) in 120 mM Universal Buffer [21] at pH = 5.0, as described elsewhere [22]. It is important to notice that all activity assays were carried out at 40°C, in order to determine the real potential of the enzymes to act at mesophilic conditions, in cold hydrolysis processes [23]. Before measuring activity of samples, kinetic profiles were constructed for all assays to guarantee that reactions were carried out under initial rate conditions. All analyses were done in triplicate. Data are expressed as mean ± 1 standard deviation (SD).

## 3. Process Model Description

The conceptual project and economic analysis of a plant for industrial-scale production of carbohydrases and proteases were done using the software SuperPro Designer version 7.5 build 8 (Intelligen Inc., Scotch Plains, NJ, USA). Auxiliary material balances were executed using Microsoft Office Excel 2003 (Microsoft Corporation, Redmond, MA, USA). Detailed information regarding the simulations will be available from the authors upon request.

### 3.1. Plant Scale

According to the Brazilian Institute of Geography and Statistics (IBGE) [24], in 2008 (most recent available data), 110,636 tons babassu coconut almond were harvested in Brazil, generating 37,616 tons of cake [25]. Since the production is concentrated in a few Brazilian states, the plant scale was defined as being able to process 94% of the total babassu cake generated in Brazil. Consequently, simulations considering different plant variables (fermentation time, amount of equipment operating in stagger mode, etc.) resulted in different amounts of the final product, that is, in different plant manufacturing capacities.

### 3.2. Costs of Raw Materials

The costs assumed for the majority of the raw materials used in the simulations were obtained from the database Aliceweb, which is hosted by the Brazilian Ministry of Development, Industry and Foreign Trade (MDIC) [26]. Average import or export FOB prices for the period from January to November 2009 were considered, depending on which shown the highest commercialization amounts. Sucrose prices, exceptionally, were obtained from the Brazilian Sugarcane Industry Association (UNICA) and calculated as the mean value of those used in the last two seasons [27]. Water cost was based on the value reported by Kwiatkowski et al. [4]. Babassu cake cost was assumed as the current practiced price, 0.3815 USD kg^−1^, which was informed by a local industry supplier. Data obtained in Brazilian currency (BRL) were converted to US Dollars using an exchange rate of 1.7299 (BRL/USD).

### 3.3. Utilities

Cooling water (*T*_in_ = 15°C, *T*_out_ = 25°C), chilled water (*T*_in_ = 5°C; *T*_out_ = 10°C), steam (*T*_in_ = *T*_out_ = 152°C), and electricity are the utilities required in the process. Their costs were considered as the default values set by the SuperPro Designer software (0.05 USD MT^−1^, 0.40 USD MT^−1^, 12.00 USD MT^−1^and 0.10 USD kWh^−1^, resp.). The demand of utilities for each unit operation, as well as for the global process, is calculated by the software.

### 3.4. Economic Analysis

The plant was scheduled to have its construction starting in 2010, for a period of 24 months, plus 1 month for start up. The total project lifetime (including construction) was set to 20 years and the annual inflation rate (4.5%) was based on the rate projected for the next years in Brazil [28]. Depreciation period was set as 18 years, assuming no further salvage value concerning the direct fixed capital (DFC) cost. According to the default method available in the software (straight line method), annual depreciation costs were equally distributed along the 18 years of depreciation. No financing was considered for the plant construction, and no expenses were assumed for product advertising. Expenses regarding R&D were set to USD 500,000 (total amount during project lifetime).

### 3.5. Capital and Operating Costs Adjustments

Based on the data reported by Kwiatkowski et al. [4], DFC of the plant was assumed as three times the total purchase cost of main equipments. Labor costs included 4 employees for technical operations (rate: 20 USD h^−1^, each) and 1 supervisor (rate: 40 USD h^−1^). Working capital was considered as the value enough to cover plant expenses corresponding to labor, raw materials, utilities, and waste treatment for 30 days of operation. The two first batches of the plant were attributed to start-up and validation tests; so their costs were taken into account in relation to the total amount of batches during the whole lifetime of the plant. Annual maintenance rate of equipments was set as 2% of DFC. The maximum annual operating period was set to 330 days.

## 4. Results and Discussion

### 4.1. Amylases and Accessory Hydrolases Production

In order to determine the process data needed for estimating throughput and costs at industrial scale, lab-scale experiments were carried out by cultivating *A. awamor*i IOC-3914 in babassu cake. In addition to endoamylases and exoamylases, which represent the target group of enzymes in this work, other groups of enzymes (cellulases, xylanases, and proteases), which are treated in this paper as accessory enzymes, were analyzed. They aggregate value to the final product stream, by synergistically contributing to the hydrolysis of starch-linked molecules (e.g., proteins), improving viscosity patterns related to degradation of polysaccharides of the hulls [11], and/or even increasing the release of sugars during a saccharification process [29].

**Figure 1:**
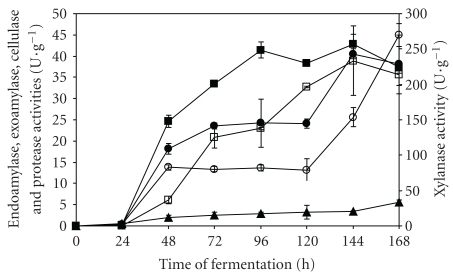
Production of amylases and accessory enzymes by SSF of babassu cake after propagation of *A. awamori* cells in starch medium: endoamylase (closed squares), exoamylase (closed circles), cellulase (open squares), protease (closed triangles), and xylanase (open circles).

The experimental profiles for the production of these enzymes, considering as base-case fungal propagation in starch medium, are presented in Figure 1. Although absolute maximum endoamylase activity had been observed after 144 hours of fermentation, considering the confidence interval of the means, it can be assumed that the production of this group of enzymes began stabilizing after 96 hours of process. Exoamylase and cellulase activities reached their maximum values after 144 hours of fermentation. Although xylanase and protease activities had continuously increased after 120 hours of incubation, experiments were stopped after 168 hours of fermentation, since the other major enzyme activities started decreasing after 144 hours. The productivity peak for endoamylase (0.43 U g^−1^ h^−1^) was observed earlier (96 hours), and thus this fermentation time was compared to 144 hours in the simulations and economic analysis.

It is important to notice that the higher xylanase activity values determined do not mean that this was the predominant group of enzymes in the extracts. The apparent superiority is due to the quantification method used, which was based on the determination of total reducing sugars (xylose and xylooligosaccharides) released after xylan hydrolysis, instead of just determining the corresponding monosaccharide, as in the exoamylase and cellulase assays [22].

### 4.2. Effect of Fermentation Time on Plant Costs and Throughput

**Figure 2:**
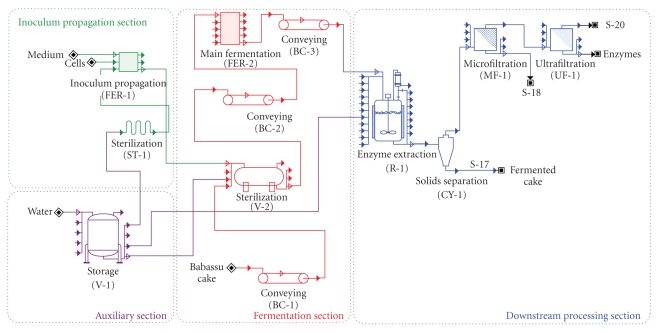
Simplified process flowsheet for the production of amylases and accessory enzymes by SSF of babassu cake.

Initial simulations were conducted for the base-case scenario (Figure 2), considering only one equipment per stage, adopting starch medium for inoculum propagation and assuming that the concentrated enzyme stream is the only revenue source (fermented cake being a waste). Therefore, streams 17, 18, and 20 in Figure 2 were treated as waste streams, with a treatment cost for disposal of 0.0005 USD kg^−1^, for each stream. All solid streams were transported using belt conveyors. Centrifugal pumps used for transporting liquid streams are not shown in the flowsheet, but were accounted for in the economic analysis. Inoculum propagation and main fermentation were conducted in tray bioreactors, as in the lab-scale experiments. After fermentation, enzymes were extracted using a CSTR and then separated from the fermented cake by a hydrocyclone, considering 80% of extract recovery. The overflow stream was directed to a microfiltration (MF) unit and finally to ultrafiltration (UF) modules for enzyme concentration. The total enzymes concentration of the UF retentate was assumed to be 50% (m/m), in order to yield a final product with a total protein concentration in the same order of magnitude as found in commercial preparations. Denaturation during these filtration procedures was assumed proportionally to all groups of enzymes and was adjusted to 5% for MF, while for UF a lower extent (2%) was used, based on experimental results reported by Mores et al. [30]. 

**Figure 3:**
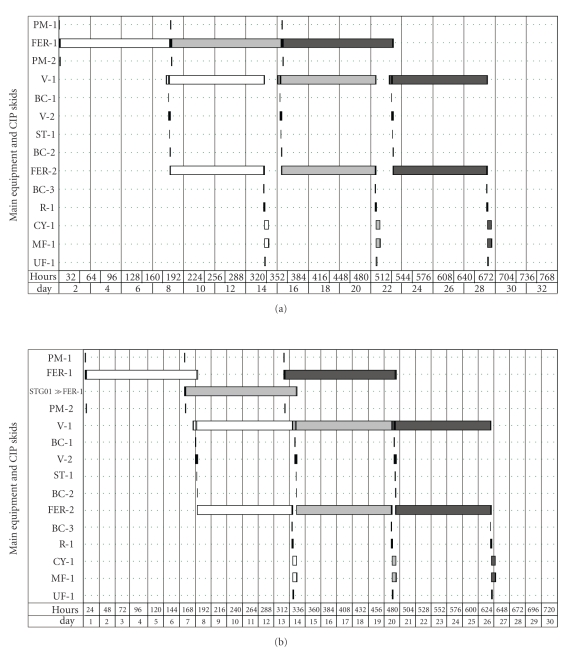
Equipment occupancy chart of an industrial plant for production of amylases and accessory enzymes considering 144 hours of fermentation, before (a) and after (b) debottlenecking analysis. White bars represent occupancy during first batch, while light grey and dark grey bars represent the occupancy during second and third batches, respectively. For abbreviations, see “list of symbols and abbreviations”.

Simulations and throughput analysis confirmed experimental observations indicating that the inoculum propagation step was a bottleneck of the process. Its total duration per batch was 172 hours (slightly more than 7 days, because its operation includes charge of nutrients, cells, water and CIP), so the other sections of the plant had idle periods, and their starting times were determined by the inoculum propagation stage, as a master-slave relationship. For this reason, a debottlenecking methodology was conducted and indicated that one extra inoculum propagation fermenter should be included in the plant (operating in a stagger mode), so that it would initiate its operation a shift time after the first inoculum propagation unit. Considering such modification, the main fermenter, which was 22 times larger than the inoculum propagation unit, had its idle time reduced from 15% to 3.6% of its total operation time per batch (Figure 3).

The insertion of one extra inoculum propagation bioreactor improved equipment occupancy, so that, as shown in Figure 3, batch duration was reduced from 666.5 hours to 626.5 hours, and the total number of batches per year was increased from 45 to 51. Since the present study was carried out considering always a constant amount of babassu cake annually processed by the plant, the higher number of batches after debottlenecking resulted in smaller equipment and, in turn, in a reduction of 19% in total capital investment. 

The equipment occupancy charts (Figure 3) also showed that among all equipment the three belt conveyors showed the highest throughput, consequently presenting idle times equal to 99.7% of their total operating times. Unfortunately, this type of equipment must operate as dedicated equipment, thus no flexibility is possible in this case. The three conveyors together contribute to 7% of total capital investment of the plant and add an annual operational cost of USD 31,800 during the first 18 years of plant operation, due to their depreciation.

To study the impact of fermentation time on the economic feasibility of the plant, a simulation considering 96 hours as fermentation time was also carried out, and then debottlenecked and compared to the previous case (144 hours of fermentation time). The goal was to investigate the impact of this change on the economic performance of the plant. As shown in Table 1, the shorter fermentation time resulted in a higher number of batches per year, a 15% decrease in total capital investment and similar operational costs, when compared to the longer fermentation time. However, according to the experimental lab-scale data presented in Figure 1, higher enzyme activities were achieved after 144 hours of fermentation; so the longer fermentation time provided a higher product throughput, resulting in a 37% decrease in the unitary production cost.

### 4.3. Effect of Inoculum Propagation Medium on Economic Indicators

*A. awamori* IOC-3914 was incubated in 5 different inoculum propagation media for 7 days, in order to determine the economically most appropriate medium for industrial scale. Based on commercialization values reported by [26, 27], the costs for production of each liter of propagation medium were calculated, and then, considering the experimental yields from the lab-scale experiments, the raw-material costs required for obtaining 10^10^ spores were calculated (Table 2). 

 Although the cheapest of the 5 propagation media was Czapeck-Dox (USD 0.30 per liter), *A. awamori* was not able to grow satisfactorily in it. A similarly poor growth behavior was observed in the base-case starch medium. Since the nitrogen source in both these media is sodium nitrate, it seems that inorganic nitrogen sources are not appropriate for the sporulation of this fungal strain.

PDA and oat media, which according to Table 2 were the most cost-effective propagation media, were then adopted in new process simulations (Table 3). The slightly higher capital investment observed for the plant using PDA propagation medium reflects the lower spores yield that was determined in the lab-scale experiments (Table 2). As a lower amount of spores is obtained per liter of medium, the prefermenter used in the propagation stage had to be larger (14% higher capacity) than the prefermenter used for obtaining spores in oat medium. Associated operating costs, on the other hand, were higher for oat medium, which is in agreement with the spores production costs calculated in Table 2.

The unitary production costs found for PDA and oat media-based processes were quite similar. For this reason, an additional criterion was included for the choice of the most suitable medium for further studies. In Brazil, oat is produced just in the Southern region, while potato, which is the major component of PDA medium, is largely produced all over the country, including in the Northeastern region, closer to states where babassu cake is generated [24]. Therefore, in order to decrease logistic costs, PDA medium was chosen for the following simulations.

### 4.4. Effect of Coproduct Credit on the Economic Performance of the Plant

As discussed above, by selecting a more appropriate inoculum propagation medium, it was possible to lower capital and operating costs. However, to evaluate if the economic performance of the plant could be further improved, the fermented cake, previously considered as a residue, was investigated as a possible process coproduct. To enable its commercialization as animal feed, an increase of 2% in cake protein content due to fermentation was assumed, based on experimental data reported by [31, 32], who worked with protein enrichment of agroindustrial materials using *Aspergillus* species. The fermented cake was, then, considered as a revenue source. *A. awamori*-derived products have GRAS status recognized by the U.S. Food and Drug Administration [33]. The selling price of the fermented cake was defined based on the 2%-increase in its protein content, so that it was estimated to be sold for 0.418 USD kg^−1^. 

 Adding a section for coproduct downstream processing increased the total capital investment almost twofold (Table 4), due to the high contact area required for tray dryers to reduce the moisture content of the cake down to 30%, and thus increase its shelf-life. However, as the amount of coproduct generated is high, the credit corresponding to this stream showed to be very significant, contributing to decrease the unitary production cost of the enzymes. Other unit operations for fermented cake drying, such as rotary drum and cone screw dryer, were also evaluated, keeping coproduct stream specifications constant, but tray dryers presented the lowest purchase cost.

### 4.5. Sensitivity Analysis

**Figure 4:**
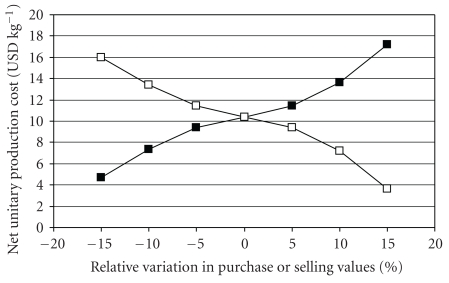
Effect of purchase price of raw babassu cake (closed symbols) and selling price of fermented babassu cake (open symbols) on the net unitary production cost of the enzyme product containing amylases and accessory enzymes. Base-case prices for raw and fermented babassu cake were, respectively, 0.38 USD kg^−1^ and 0.42 USD kg^−1^.

Babassu cake, the primary feedstock, presented the greatest influence on production cost of the enzyme product. Such raw material has its selling price regulated by other cakes with significantly higher commercialization amounts, such as soy cake, being subject to price variations from one season to another. Also, for the animal feed market, the feed price is directly proportional to its protein content. Therefore, sensitivity analyses were carried out in order to investigate the effects of variations in the purchase price of raw babassu cake and in the selling prices of fermented cake on the net unitary production cost of enzymes. According to business information of the babassu industry, depending on the demand and under favorable conditions, the selling price of raw babassu cake could reach a minimum of 0.25 USD kg^−1^. Thus, the currently practiced price was varied over a range of ±15% on its base-case price (0.38 USD kg^−1^), as shown in Figure 4. It can be observed that the most significant influence on the unitary production cost would be due to an increased selling price of fermented cake, which could be achieved, for example, if the *Y*_*X*/*S*_ of the fermentative step were increased. Moreover, it is important to notice that a minor portion of the enzymes produced remained within the fermented cake after the enzyme extraction step, so contributing for an increase in the cake protein content.

A similar sensitivity analysis was done by Kwiatkowski et al. [4] for the production of ethanol from corn. The authors found that corn corresponded to 88.5% of the total raw materials cost and an increase of 77% in the corn price elevated the unitary production cost of ethanol from 0.24 USD L^−1^ to 0.37 USD L^−1^. In the present work, considering the final process configuration, raw babassu cake contributed to approximately 90% of the total raw materials costs, corresponding to 60% of the total operational costs of the plant.

The impact of the coproduct credit on unitary production cost of the enzyme product (and consequently on final selling price) was confirmed to be very significant. Depending on the market conditions, revenues with sales of fermented cake can turn the production cost of enzymes stream as low as 3.66 USD kg^−1^, and thus the main product can be sold by a very competitive price. An economic analysis of the corn dry-grind ethanol process that made elsewhere, also using SuperPro Designer, revealed that credits from commercialization of the coproduct DDGS increased the plant revenues by 27%, improving plant profitability [4].

Perkis et al. [13] reported that endoamylase-based and exoamylase-based products for application in the ethanol industry present sales prices of 12.1 USD kg^−1^ and 6.95 USD kg^−1^, respectively. The concentrated enzyme stream which form the product of the plant simulated in the present work is considered as a high-value product due to two major aspects: the presence of accessory enzymes (cellulases, xylanases, and proteases), which were previously pointed out as important biocatalysts to improve ethanol yield in the corn-to-ethanol process [12, 13]; and the capacity of enzymes to act at low temperatures (below 40°C), thus allowing cold hydrolysis of polysaccharides. Cold hydrolysis has been discussed recently in the literature as a possible technological trend in the starchy ethanol industry [7–9] and as a great alternative over the traditional processes that employ very high temperatures for cooking and liquefaction, and relatively high temperature for saccharification [4, 23]. Therefore, the net unitary production cost (10.4 USD kg^−1^) of the enzyme product discussed in the present work compares well with the prices reported by Perkis et al. [13], considering that the present enzyme product could be commercialized by higher prices due to their characteristics that allow it to be used in cold-hydrolysis processes.

## 5. Conclusions

The present study combined solid-state fermentation experiments with simulations using the software SuperPro Designer to investigate the production of fungal mesophilic enzymes, aiming at their use in cold hydrolysis processes for bioethanol production.

The combination of experiments and simulations indicated that 144 hours is the most appropriate fermentation time for producing an amylolytic complex enriched by accessory enzymes (cellulases, xylanases, and proteases). The inoculum propagation step showed to be the limiting step of the process, both in terms of raw material costs and plant schedule (impacting on equipment occupancy and size). To tackle the latter, debottlenecking tools available in the software were used to investigate the best process configuration to improve equipment occupancy and decrease production costs. To tackle the issue of raw material costs, five different inoculum propagation media were evaluated experimentally and, based on the spores yield and costs of each of them, the two most promising propagation media (PDA and oat medium) were evaluated in further simulations with the software. 

 Simulations using the final, debottlenecked process, employing PDA medium as the most appropriate propagation medium, showed that commercialization of the fermented cake generated after enzyme extraction for animal feed applications is important to enable a greater decrease in the selling price of the main enzyme product. Thus, considering the fermented cake as coproduct, the unitary production cost of the enzyme product was 10.40 USD kg^−1^. This is a competitive price for this product due to its advantageous characteristics, such as the presence of desirable accessory hydrolytic enzymes and the ability to hydrolyze raw starch at mild mesophilic conditions, compatible with cold hydrolysis processes.

## Figures and Tables

**Table 1:** Effect of fermentation time on economic and throughput indicators of the enzyme production plant (both cases shown after debottlenecking).

Economic/throughput indicator	Fermentation time (h)	
	96	144
Batches per year	74	51
Annual product throughput (ton of product stream)	353.36	573.30
Total capital investment (10^6^ USD)	22.40	26.04
Annual operating cost (10^6^ USD)	19.32	19.58
Unitary production cost (USD kg^−1^ of product stream)	54.66	34.16

**Table 2:** Effect of inoculum propagation medium on yield and costs of production of *A. awamori* IOC-3914 spores.

Inoculum propagation medium	Yield (10^8^ spores L^−1^)	Cost per 10^10^ spores (USD)
Czapeck-Dox	3.7 ± 1.9	0.16 ± 0.08
Malt	4.0 ± 1.4	0.30 ± 0.11
PDA	28.0 ± 0.5	0.04 ± 0.00^(a)^
Starch	4.8 ± 0.8	0.19 ± 0.03
Oat	32.3 ± 0.1	0.05 ± 0.00^(a)^

^(a)^Standard deviation values were unequal zero, but below 0.01 USD.

**Table 3:** Effect of inoculum propagation medium on the economic performance of a plant for production of amylases and accessory enzymes.

Parameter	PDA medium	Oat medium
Total capital investment (10^6^ USD)	21.64	21.50

Itemized operating costs (10^3^ USD)		

Raw materials	13,981	14,263
Utilities	526.00	527.00
Waste treatment and disposal	276.00	277.00
Unitary production cost (USD kg^−1^ of product stream)	26.01	26.42

**Table 4:** Economic evaluation of a debottlenecked process for production of amylases and accessory enzymes using PDA propagation medium, after 144 hours of fermentation, considering coproduct credits due to fermented cake commercialization.

	Value
Total capital investment (USD)	42,272,837
Total annual operating cost (USD)	19,922,736
Annual throughput for enzymes stream (kg)	644,125
Annual throughput for fermented cake stream (ton)	31,668
Coproduct credit (USD)	13,225,825
Net unitary production cost (USD kg^−1^ of product stream)	10.40

## List of Symbols and Abbreviations

BC-*n*: Belt conveyor for transport of solids (where *n* = 1, 2, 3)
CIP: Cleaning in place
CSTR: Continuous stirred-tank reactor
CY-1: Hydrocyclone for separation of enzyme extract from fermented cake
DDGS: Distillers' dried grain with solubles
DFC: Direct fixed capital cost of the plant. Includes direct costs (piping, instrumentation, insulation, electrical facilities, buildings, yard improvements and auxiliary facilities), indirect costs (engineering and construction) and other costs (contractor's fee and contingency).
FER-1: Prefermenter for inoculum propagation
FER-2: Main fermenter for production of amylases and accessory hydrolases
FOB: Free-on-board price
MF-1: Microfiltration unit for separation of fungal cells from enzyme extract
MT: Metric ton
NPV: Net present value
PDA: Potato-dextrose agar medium
R-1: CSTR for enzyme extraction from fermented solids
R&D: Research and development
S: Denotes a stream
SmF: Submerged fermentation
SSF: Solid-state fermentation
ST-1: Water sterilization unit serving the prefermenter
STG01: Indicates equipment operating in stagger mode
*T*_in_: Inlet temperature, °C
*T*_out_: Outlet temperature, °C
UF-1: Ultrafiltration unit for concentration of enzymes
V-1: Water storage tank
V-2: Sterilization drum for babassu cake and moisture adjustment
VOC: Volatile organic compounds
*Y*_*X*/*S*_: Cell growth yield coefficient
GRAS: Generally recognized as safe.
